# Individual and Interpersonal Factors Associated with the Incidence, Persistence, and Remission of Internet Gaming Disorders Symptoms in an Adolescents Sample

**DOI:** 10.3390/ijerph182111638

**Published:** 2021-11-05

**Authors:** Rosario J. Marrero, Ascensión Fumero, Dolores Voltes, Manuel González, Wenceslao Peñate

**Affiliations:** Department Clinical Psychology, Psychobiology and Methodology, Universidad de La Laguna, 38205 San Cristóbal de La Laguna, Spain; afumero@ull.edu.es (A.F.); lolavoltes@gmail.com (D.V.); mgonzaro@ull.edu.es (M.G.); wpenate@ull.edu.es (W.P.)

**Keywords:** gaming disorder, emotional problems, family adjustment, personality, impulsivity, hostility, adolescence

## Abstract

Video game playing behavior has serious consequences for adolescents on a personal, family, social, and academic level. This research aimed to examine risk and protective factors involving incidence, persistence, and remission of gaming disorders symptoms (IGDs) in Spanish adolescents after nine months of follow-up. Data were drawn from self-administered questionnaires completed on two occasions: at the beginning (T1) and end of the academic year (T2). A total of 950 adolescents aged from 11 to 20 years (M = 14, SD = 1.52, 48.5% female) completed the questionnaire at T1, while 550 adolescents aged from 11 to 18 years (M = 13.43, SD = 1.23, 48.9% female) took part in the follow-up study (T2). The incidence, persistence, and remission rates were 6%, 2.7%, and 4.2%, respectively. Significant relationships with IGDs were found between the male gender and studying at a private school in T1 and T2. The findings show that the time spent playing video games during T1 was positively associated with IGDs in T1 and T2. The incidence of IGDs was associated with emotional problems and low family affection. The persistence of IGDs was linked to higher motor impulsivity, agreeableness, and lower family resolve. Remission was related to a decrease in anxiety and hostility as well as an increase in the emotional stability of adolescents. These findings imply that emotional well-being and family adjustment could be relevant for the effective management of gaming behaviors.

## 1. Introduction

Behavioral addictions constitute a serious problem in childhood and adolescence, with significant repercussions on the physical, psychological, and social health of those involved and the people around them. Adolescence is a critical period not only due to the changes that occur at the brain-maturational level that make adolescents more vulnerable to mental health problems [1] but also because the context can exert a greater influence on gaming behavior, reinforcing the processes of imitation [2]. Thus, more frequent contact with new technologies and peers can lead to more personal and social dangers, which become even more negative during adolescence.

Internet gaming disorder (IGD) has been included in the Diagnostic and Statistical Manual of Mental Disorders (DSM-5) as a health problem that needs further research [3]. However, the 11th revision of the International Classification of Diseases (ICD-11) [4] introduces the term “Gaming Disorders” under mental disorders, defining it as a pattern of video game playing behavior characterized by poor control of such behavior, which prioritizes gaming over other activities and increases gaming despite the occurrence of negative consequences, leading to clinically significant deteriorations over a period of 12 months [3,4]. Currently, there is no agreement among theorists, researchers, and clinicians about whether video game playing behavior can be considered an addiction or an abusive behavior that remits with age [5,6,7]. Hence, in the current study, the term Internet gaming disorder symptoms (IGDs) has been used instead of Internet gaming disorder, along with the fact that the self-report measure does not provide information about symptoms presentation in the previous 12 months.

The prevalence of abusive video game behavior ranges from 0.3% to 27.5% [8], the worldwide prevalence being around 3.05% [9]. In Spain, it ranges from 1.9% to 8% [10]. IGDs have shown a higher prevalence in boys [11]. Most studies report that boys have a 2–3 times greater risk of Internet gaming than girls [12]. Previous research shows the existence of a high degree of comorbidity between IGDs and emotional disorders, obsessive-compulsive disorder, attention deficit hyperactivity disorder, and substance abuse [13]. In addition, comorbidity has been observed with externalizing behavior disorders characterized by hostility/aggressiveness [14] and with social phobia [15].

This comorbidity between different disorders has also been explained as a function of possible shared neuro-physiological correlates. Functional magnetic resonance imaging (fMRI) studies have shown that during gaming, there is an activation of the brain areas involved in the reward system, such as the right dorsolateral prefrontal cortex, bilateral premotor cortices, right insula, precuneus, and areas involved in motor function and visual processing [16]. Neuroimaging studies on gaming in adolescents show alterations in the prefrontal cortex leading to imbalance of cognitive control. Some studies suggest alterations in temporoparietal areas that could affect attentional problems. In addition, frontolimbic regions, such as the orbitofrontal cortex and amygdala, seem to be involved in poor reactions toward negative emotions and problems in emotion regulation. Moreover, a higher global and lower local neural efficiency have been found in adolescents with gaming disorders, which can lead to imbalance between segregation and integration and less efficiency in control processes. Additionally, problems of interhemispheric connectivity of prefrontal areas have been found in adolescents with gaming disorders [1].

Risk factors associated with IGDs have been identified, including individual, interpersonal, psychosocial, and contextual factors [17,18]. At the individual level, impulsivity, aggressiveness, hostility, sensation seeking, anxiety, depression, or social withdrawal have been associated with IGDs [11,19]. In addition, high neuroticism, low conscientiousness, low extraversion, and low agreeableness have been linked to IGDs and Internet use disorder [20,21,22], the conscientiousness trait being the one that mainly shows the protective role of the online game [23]. The most relevant interpersonal factors associated with IGDs have been problematic family relationships, family cohesion difficulties, parents’ mental health problems, and the absence of rules for Internet game use [11]. Contextual factors associated with IGDs include accessibility to the Internet, affordability of different devices, and time spent engaging in the problematic behavior [24,25]. The role of time spent playing video games on IGDs has been controversial. On the one hand, time spent playing video games has been associated with sleep loss [26], poor academic performance [27], perceived isolation [28], and mood [15]. On the other, the amount of time spent gaming is not predictive, and no association was found with negative consequences at the social, emotional, and psychological level [29].

The protective factors for IGDs included personal characteristics, such as self-esteem, self-identity, self-efficacy, or interpersonal well-being [30]. Positive family factors, including positive perceptions of the family environment, warmth in the family environment, or closeness between parents and children, also function as a protector against video game addiction [31].

Longitudinal studies showed controversial results regarding the incidence of IGDs [8]. Some authors place the incidence between 4.9% and 7.7% after one year [14,32], while others indicate that these rates remain stable over long periods [33,34]. Additionally, some studies indicate both an increasing and decreasing trend in symptom severity across time [35,36]. Moreover, greater severity of IGD is associated with losing interest in previous activities and losing one’s job, studies, or career opportunity because of playing [37]. Sociodemographic variables, such as male gender or a poor family financial situation, as well as considerable time spent on gaming and depressive symptoms were related to IGD incidence after one year [32]. Regarding risk factors associated with long-term disorder, adolescents with clinical IGDs have been found to exhibit higher levels of depression, anxiety, impulsivity, and social phobia during the follow-up period than moderate adolescent gamers [15,38]. Other longitudinal studies indicated that low social competence, lower self-esteem, higher extraversion, and greater loneliness predicted addiction over time [39,40]. Other factors involved in the development of IGDs include a positive attitude toward gaming and the intention to play [41], substances consumption, aggression [42], behavioral problems, poor academic performance [43], hyperactivity or inattention [44], and excessive parental psychological control [45].

Most of the studies have focused on analyzing the factors involved in the incidence of IGDs. However, there is a differential development pattern over time both in Internet addiction [46,47] and in gaming [36]. On the one hand, there are adolescents who do not show IGDS but become new cases after a period of time (incidence); other adolescents who maintain IGDs from baseline to follow-up (persistence); those who stop playing after a period of time (remission); and those who did not present IGDs at any time (without IGDs). Change of predictors is observed in the incidence or remission of Internet addiction [47]. Incidence was associated with higher depression and hostility, whereas remission is associated with lower depression, hostility, and social anxiety [47]. In the same vein, Internet addiction predictors differ for incidence and persistence [46]. Persistence was associated with greater depressive symptoms and maternal education. Incidence, however, was related to low family income, being an only child, being male, and school maladjustment. Changes have also been found in gaming predictors depending on incidence, persistence, and IGD severity [36]. Incidence was associated with longer playing time per day on weekdays, playing multi-player games, being male, attention deficit hyperactivity disorder (ADHD) and depression symptomatology, and non-intact families. Persistence was associated with longer playing time, being male, and ADHD symptomatology. IGD severity was related to higher playing time, single-play, and higher anxiety trait [36].

In recent years, several cross-sectional and longitudinal studies have analyzed the psychosocial and contextual factors involved in IGDs. However, there is still some controversy about the relative weight of individual (e.g., deficient self-regulation) and interpersonal factors (e.g., difficulties in family functioning) associated with IGD in adolescents [17,30]. A research gap is the simultaneous analysis of these individual and interpersonal factors, which could act as risk or protective factors, in adolescents who start gaming (incidence), continue to play over time (persistence), and stop playing after a period of time (remission).

The purposes of the current study were: (1) to identify the incidence, persistence, and remission rate of IGDs in Spanish adolescents after nine months of follow-up. It was hypothesized that IGD incidence will increase, decrease, or remain stable during a nine-month follow-up period [14,32,33,36]; (2) to analyze the individual, interpersonal, and contextual factors associated with IGDs in T1 and T2. A positive relationship between depression, anxiety, impulsivity, hostility, being a man, time spent playing video games and IGDs, and a negative relationship between emotional stability, conscientiousness, extraversion, openness to experience, agreeableness, family functioning, and IGDs is expected in both T1 and T2 [11,15,25]; (3) to examine individual and interpersonal factors for adolescents who developed IGDs (incidence), those who maintained IGDs (persistence), and those who had remission of IGDs (remission) on two separate occasions over nine-month follow-up periods. It was hypothesized that there will be an increase in anxiety, depression, impulsivity, and hostility [19,38] as well as low emotional stability, conscientiousness, extraversion and agreeableness [21], and difficulties in family functioning [11] in adolescents who initiate and maintain IGDs, while an opposite effect is expected for adolescents in whom IGDs have remitted [36]. However, it is also expected that there may be a change in the individual and interpersonal factors that will be associated with the different development pattern in the course of IGDs; and (4) to estimate risk and protective factors on the first occasion (T1) that predict the IGDs on the second occasion (T2). It is hypothesized that individual factors at T1 (personality, hostility, impulsivity, and emotional problems) will predict high risk of IGDs at T2, and the interpersonal factors at T1 (family functioning) will predict high protection to IGDs at T2 [32,48].

## 2. Materials and Methods

### 2.1. Participants

In the current study, we assessed a convenience sample of 950 middle school students who were aged from 11 to 20 years (M = 14, SD = 1.5, 48.5% female) and who were residents in Las Palmas de Gran Canaria (Spain). Although the participants resided in a specific location, they shared cultural and social values with adolescents from the rest of Spain. In fact, in a national survey on video gaming use in young people, it was found that the rates of video game use have been shown to be similar with 39.6% of young people in the Canary Islands showing gaming behavior and 44.1% across the country [49]. Of this global sample, only 1% was over 18 years old, and of these, one young person was 20 years old. From T1 to T2, 400 adolescents (42.1%) dropped out of the study. Nonresponse was attributable to entire classes dropping out due to internal scheduling problems at T2.

The final sample was 550 adolescents from 11 to 18 years (M = 13.4; SD = 1.2, 48.9% female) in T1. Of the 550 adolescents, only 2 (0.4%) were 18 years old, and the remaining 97.6% were under 16 years of age. Age was not recorded in T2 since only 9 months had elapsed since the first wave. Fifty percent studied at public schools and 50% at private schools. A total of 74.2% came from nuclear families living with their father, mother, and in some cases siblings, while 18.6% lived in single-parent households, and 7.2% lived in reconstituted families.

### 2.2. Instruments

Participants provided sociodemographic data on gender, age, type of school, family structure, and approximate daily time in minutes spent on video gaming during the week and on weekends.

Ten Item Personality Inventory—TIPI [50]. This inventory contains ten items to assess the five major personality dimensions: extraversion (e.g., extraverted, enthusiastic), agreeableness (e.g., sympathetic, warm), conscientiousness (e.g., dependable, self-disciplined), emotional stability (e.g., anxious, easily upset), and openness to experience (e.g., open to new experiences, complex) [51]. Each dimension is measured by the mean of two items rated on a seven-point Likert-type scale ranging from 1 (strongly disagree) to 7 (strongly agree). Higher scores represent greater presence of that personality trait. The inventory has demonstrated adequate psychometric properties [52]. In this study, Cronbach’s alpha was 0.54. To evaluate the reliability of the instruments at time 1 and time 2, the intra-class correlation coefficients (ICC), using a two-way mixed effects model and type consistency, were used. ICC for the total scale was 0.53 (95%CI = 0.47 to 0.59) at T1 and 0.60 (95%CI = 0.54 to 0.65) at T2. The test-retest reliability for each subscale was also calculated through the intra-class correlation coefficient (ICC), using a two-way mixed-effects model and type consistency. The ICC values were 0.62 (95%CI = 0.56 to 0.68) for extraversion, 0.60 (95%CI = 0.53 to 0.66) for agreeableness, 0.58 (95%CI = 0.51 to 0.65) for conscientiousness, 0.60 (95%CI = 0.52 to 0.66) for emotional stability, and 0.53 (95%CI = 0.44 to 0.60) for openness to experience.

The Spanish validated version [53] of the Hospital Anxiety and Depression Scale—HADS [54] was used to measure anxiety and depression. This scale is composed of 14 items, seven for anxiety (e.g., I feel tense or “wound up”) and seven for depression (e.g., I feel as if I am slowed down). It is a Likert-type scale ranging from 0 (nothing/never) to 3 (a lot). The Cronbach’s alpha of the Spanish version was 0.90 [55]; in this study, it was 0.70. ICC for the total scale was 0.71 (95%CI = 0.67 to 0.74) at both T1 and T2. The ICC values for the anxiety and depression subscales over the test-retest interval were 0.71 (95%CI = 0.66 to 0.75) and 0.61 (95%CI = 0.53 to 0.67), respectively.

Problematic Videogame Playing—PVP [56] is a dichotomous, nine-item instrument that assesses the pathological use of online video games and is applied to measure IGDs. The items refer to the nine diagnostic criteria according to DSM-5: (1) excessive preoccupation with Internet games (e.g., when I am not playing with the video games, I keep thinking about them, i.e., remembering games, planning the next game, etc.), (2) withdrawal symptoms (e.g., when I can’t use the video games, I get restless or irritable), (3) unsuccessful attempts to control playing (e.g., I have tried to control, cut back, or stop playing, or I usually play the video games over a longer period than I intended), (4) Internet gaming becoming a dominant daily activity despite awareness of its psychosocial consequences (e.g., in order to play video games, I have skipped classes or work, or lied, or stolen, or had an argument or a fight with someone), (5) tolerance refers to the need to spend increasing amounts of time engaged in Internet games (e.g., I spend an increasing amount of time playing video games), (6) use of Internet games to escape or weaken negative moods (e.g., when I feel bad, e.g., nervous, sad, or angry, or when I have problems, I use the video games more often), (7) interpersonal problems with friends and family due to video games (e.g., because of the video game playing, I have reduced my homework, or schoolwork, or I have not eaten, or I have gone to bed late, or I spent less time with my friends and family), (8) loss of interest in previous hobbies and leisure activities (e.g., sometimes I conceal my video game playing from the others, that is, my parents, friends, teachers), and (9) risking or losing one’s job or training/career opportunity because of playing (e.g., when I lose a game or I have not obtained the desired results, I need to play again to achieve my target) [2]. In this study, the participants who replied affirmatively to five or more of the nine items were classified as having IGDs, following the DSM-5 IGD diagnosis suggestions. Cronbach’s alpha of the original instrument was 0.69 [56]; in this study, it was 0.68. ICC for the total scale was 0.68 (95%CI = 0.64 to 0.72) at T1 and 0.72 (95%CI = 0.68 to 0.75) at T2. The ICC value over the test-retest interval for PVP was 0.74 (95%CI = 0.70 to 0.78).

The Spanish validated version of the Symptom Checklist—90—Revised (SCL—90—R) [57] was used to measure hostility [58]. The eight-item checklist (e.g., feeling easily annoyed or irritated) has a five-point Likert scale ranging from 0 (total absence of the symptom) to 4 (greater intensity of the symptom), with a Cronbach’s alpha of 0.81. In this study, Cronbach’s alpha was 0.84. ICC for the total scale was 0.85 (95%CI = 0.82 to 0.87) at T1 and 0.87 (95%CI = 0.86 to 0.89) at T2. The ICC value over the test-retest interval for hostility subscale was 0.72 (95%CI = 0.66 to 0.76).

The Spanish validated version [59] of the Family APGAR [60] was used to measure family functioning. Respondents rate five items on a three-point scale of 0 (almost never), 1 (sometimes), and 2 (almost always). The items refer to adaptability (the use of intra- and extra-familial resources to solve problems in situations of family stress or crisis, e.g., I am satisfied with the help that I receive from my family when something is troubling me), partnership (the sharing of decision making and nurturing responsibilities by family members, e.g., I am satisfied with the way my family discusses items of common interest and shares problem solving with me), growth (the development of physical and emotional maturation and self-fulfillment achieved by family members through mutual support and guidance, e.g., I find that my family accepts my wishes to take on new activities or make changes in my lifestyle), affection (the caring or loving relationship that exists among family members, e.g., I am satisfied with the way my family expresses affection and responds to my feelings, such as anger, sorrow, and love), and resolve (the commitment to spend time with other family members for physical and emotional nurturing and the decision to share wealth and space, e.g., I am satisfied with the amount of time my family and I spend together). The Cronbach’s alpha was 0.84 [59], while in this study, it was 0.66. ICC for the total scale was 0.67 (95%CI = 0.62 to 0.71) at T1 and 0.71 (95%CI = 0.67 to 0.75) at T2. The ICC value over the test-retest interval for the total score for the APGAR was 0.69 (95%CI = 0.64 to 0.74).

The brief Spanish validated version (BIS–15S) [61] of the Barratt Impulsiveness Scale—BIS [62] was used to measure impulsiveness. Its 15 items are answered on a four-point scale ranging from 1 (rarely/never) to 4 (almost always/always). The scale includes three subscales: motor impulsivity (acting without thinking, e.g., I do things without thinking), attentional impulsivity (making quick cognitive decisions, e.g., I don’t pay attention), and non-planning impulsivity (lack of concern about the future, e.g., I say things without thinking). A total impulsivity score was obtained by adding the three dimensions. The Cronbach’s alpha was 0.79 [61]; in this study, it was 0.75. ICC for the total scale was 0.76 (95%CI = 0.73 to 0.79) at T1 and 0.77 (95%CI = 0.74 to 0.80) at T2. The ICC values over the test-retest interval for motor impulsivity, non-planning impulsivity, and attentional impulsivity were 0.74 (95%CI = 0.69 to 0.79), 0.55 (95%CI = 0.46 to 0.62), and 0.74 (95%CI = 0.69 to 0.78), respectively.

### 2.3. Procedure

Participants were enrolled from several secondary education centers between September 2016 and June 2017. The battery of questionnaires was applied in the classroom, guided by trained teachers and one of the researchers. In cases where participants expressed any doubts about the items, these were clarified. Each participant used an identification code consisting of the initials of their first and last name and a randomly assigned number that they had to write down in order to match their answers with those given in the second wave and to maintain anonymity. The survey took about 45 min to complete during each wave. The nine-month longitudinal study involved filling out the questionnaires on two occasions: at the beginning (T1) and end of the academic year (T2). Written informed consent was obtained from the participants and their parents before inclusion and conducted in compliance with the Helsinki Declaration. The researchers adopted the principles contained in the Code of Good Practices of University of La Laguna (OF002/2018/6521).

### 2.4. Statistical Analysis

First, the differences between the participants who took part in T1 and T2 and those who dropped out of the study were analyzed using chi-square tests and Analyses of Variance (ANOVAs). Second, descriptive statistics were calculated to describe the sample across T1 and T2. Third, correlational analyses were obtained between IGDs, sociodemographic variables, and remaining variables in both T1 and T2. Fourth, a Multivariate Analysis of Covariance (MANCOVA) of repeated measures was used with one within-subject factor (T1 and T2) and one between-group factor (groups with and without IGDs), with gender and school type as covariates. The main effects of group and of time as well as the interaction of group and time were analyzed in this way. Simple effects through two-way repeated measure ANCOVAs were then conducted to examine the between-group comparison at T1 and T2. Fifth, to identify the changes in IGDs and in individual and interpersonal variables over time (T1 vs. T2) for each group of adolescents (incidence group, persistence group, and remission group), paired sample *t*-test analyses were performed. The incidence was analyzed by identifying the young people who did not present IGDs in T1 but developed symptoms in T2. Next, the persistence rates were analyzed identifying the young people who presented IGDs in both T1 and T2. Lastly, remission was analyzed by identifying the group of young people who had IGD symptoms in T1 but did not show these symptoms in T2. Finally, a logistic regression analysis that included personal and family variables from T1 was carried out to identify the main predictors of IGDs at T2, controlling for gender and type of school. The data were analyzed using version 25 of the SPSS statistical software (IBM; New York, NY, USA).

## 3. Results

### 3.1. Descriptive Statistics and Preliminary Correlations

ANOVAs revealed that adolescents who dropped out between T1 and T2 were older (χ^2(1)^ = 214.26, *p* < 0.001) and were enrolled in public schools (χ^2(1)^ = 108.29, *p* < 0.001). Additionally, they had higher scores on anxiety (F(1, 678) = 82.62, *p* < 0.001, η^2^ = 0.11), depression (F(1, 678) = 325.64, *p* < 0.001, η^2^ = 0.32), hostility (F(1, 678) = 11.82, *p* < 0.001, η^2^ = 0.02), motor impulsivity (F(1, 678) = 30.04, *p* < 0.001, η^2^ = 0.04), and attentional impulsivity (F(1, 678) = 12.76, *p* < 0.001, η^2^ = 0.02). They also showed lower scores on extraversion (F(1, 678) = 11.24, *p* < 0.001, η^2^ = 0.02), agreeableness (F(1, 678) = 12, *p* < 0.001, η^2^ = 0.02), conscientiousness (F(1, 678) = 19.54, *p* < 0.001, η^2^ = 0.03), emotional stability (F(1, 678) = 11.25, *p* < 0.001, η^2^ = 0.02), openness to experience (F(1, 678) = 9.66, *p* < 0.01, η^2^ = 0.01), family adaptation (F(1,678) = 19.50, *p* < 0.001, η^2^ = 0.03), family growth (F(1, 678) = 5.51, *p* < 0.05, η^2^ = 0.008), family partnership (F(1, 678) = 3.98, *p* < 0.05, η^2^ = 0.006), and family resolve (F(1, 678) = 15.71, *p* < 0.001, η^2^ = 0.02) although effects were small in most of the variables. No significant differences in gender (χ^2(1)^ = 0.08, *p* = 0.78) or family type (χ^2(2)^ = 0.11, *p* = 0.945) on the problematic gaming measure (F(1, 678) = 1.25, *p* = 0.264), family affection (F(1, 678) = 2.66, *p* = 0.103), or non-planning impulsivity (F(1, 678) = 0.78, *p* = 0.378) were found.

Table 1 shows the descriptive statistics and the correlations of the different variables included in the longitudinal study for final sample (*n* = 550). The mean IGDs score at T1 was 1.58 (SD = 1.75) and, at T2, 1.54 (SD = 1.77). Of the total sample participating in the research, 37 students in T1 (6.73%) and 48 students in T2 (8.73%) fit the IGDs criterion (greater than or equal to 5 points). In T1, the mean time spent playing video games during the week was 33.86 min (SD = 70.39) and 117.77 min (SD = 153.94) at the weekend.

In both T1 and T2, a positive relationship was found between IGDs with gender and type of school. Males and private school students showed higher IGDs. Furthermore, IGDs were also associated with greater emotional symptoms, motor and attentional impulsivity, and hostility. Negative relationships appeared from IGDs with agreeableness and conscientiousness as well as with family adaptability and partnership. The time spent on video games during the week of T1 was positively associated with the IGDs of T1 (r = 0.28, *p* < 0.001) and T2 (r = 0.30, *p* < 0.001). Similarly, playing time on the weekend of T1 was linked to the IGDs of T1 (r = 0.50, *p* < 0.001) and T2 (r = 0.59, *p* < 0.001). The pattern of correlations between the remaining variables included in the study was similar in T1 and T2. The highest magnitude relationships were between anxiety with depression (r = 0.32, *p* < 0.001 in T1; r = 0.46, *p* < 0.001 in T2), hostility (r = 0.48, *p* < 0.001 in T1; r = 0.52, *p* < 0.001 in T2), and attentional impulsivity (r = 0.39, *p* < 0.001 in T1; r = 0.42, *p* < 0.001 in T2). In addition, high significant relationships were found between hostility with motor impulsivity (r = 0.55, *p* < 0.001 in T1; r = 0.54, *p* < 0.001 in T2) and attentional impulsivity (r = 0.45, *p* < 0.001 in T1 and T2). Lastly, negative associations were found between emotional stability and hostility (r = −0.44, *p* < 0.001 in T1; r = −0.41, *p* < 0.001 in T2), motor impulsivity (r = −0.42, *p* < 0.001 in T1; r = −0.38, *p* < 0.001 in T2), and attentional impulsivity (r = −0.39, *p* < 0.001 in T1; r = −0.40, *p* < 0.001 in T2).

### 3.2. Between-Group and within-Subject Analyses

The MANCOVA, controlling for gender and school, in which the scores in the different variables assessed in T1 and T2 were compared (within-subject) for both groups with and without IGDs in T2 (between-groups), showed there were no significant effects for the group x time interaction (Table 2). Simple effects of time were found for anxiety and marginally significant for depression and motor impulsivity. Simple effects of groups were found for anxiety, depression, agreeableness, conscientiousness, emotional stability, motor impulsivity, attentional impulsivity, hostility, and family functioning (adaptability, growth, resolve, and affection).

In T1, significant differences in most of the variables assessed between both with and without IGDs groups were found. Specifically, the IGDs group scored higher in anxiety (F(1, 546) = 15.76, *p* < 0.001, η^2^ = 0.03), depression (F(1, 546) = 17.76, *p* < 0.001, η^2^ = 0.03), motor impulsivity (F(1, 546) = 7.28, *p* < 0.01, η^2^ = 0.01), attentional impulsivity (F(1, 546) = 15.73, *p* < 0.001, η^2^ = 0.03), and hostility (F(1, 546) = 15.02, *p* < 0.001, η^2^ = 0.03) and lower in agreeableness (F(1, 546) = 27.95, *p* < 0.001, η^2^ = 0.05), conscientiousness (F(1, 546) = 11, *p* < 0.001, η^2^ = 0.02), emotional stability (F(1, 546) = 20.73, *p* < 0.001, η^2^ = 0.04), and openness to the experience (F(1, 546) = 4.29, *p* < 0.05, η^2^ = 0.00) than the group without IGDs. In addition, the IGDs group showed lower family adaptability (F(1, 546) = 11.03, *p* < 0.001, η^2^ = 0.01) and partnership (F(1, 546) = 9.98, *p* < 0.01, η^2^ = 0.00) compared to the group without IGDs.

In T2, the IGDs group scored higher on anxiety (F(1, 546) = 13.46, *p* < 0.001, η^2^ = 0.02), depression (F(1, 546) = 17.69, *p* < 0.001, η^2^ = 0.03), motor impulsivity (F(1, 546) = 16.31, *p* < 0.001, η^2^ = 0.03), attentional impulsivity (F(1, 546) = 14.05, *p* < 0.001, η^2^ = 0.03), hostility (F(1, 546) = 13.65, *p* < 0.001, η^2^ = 0.02), agreeableness (F(1, 546) = 10.88, *p* < 0.001, η^2^ = 0.02), conscientiousness (F(1, 546) = 12.05, *p* < 0.001, η^2^ = 0.02), and emotional stability (F(1, 546) = 2.82, *p* < 0.10, η^2^ = 0.01). In addition, the IGDs group was characterized by showing less family affection (F(1, 546) = 4.86, *p* < 0.05, η^2^ = 0.01) compared to those without IGDs.

### 3.3. Difference of Means in the Variables Evaluated According to the Incidence, Persistence, and Remission of IGDs

Three groups were identified based on the presence or absence of IGDs in the course of the nine months: incidence group 1 was made up of 33 students (6% of the sample) without IGDs in T1 and those who developed IGDs in T2 (81.8% boys, *n* = 27); persistence group 2 was made up of 15 students (2.7% of the sample) with IGDs in both T1 and T2 (73.3% boys, *n* = 11); and remission group 3 was made up of 23 students (4.2% of the sample) with IGDs in T1 and without IGDs in T2 (82.6% boys, *n* = 19). Table 3 gives the mean differences for the IGDs incidence group. These students showed a significant increase in IGDs levels, higher levels of anxiety and depression, and lower scores in family affection in T2 than T1.

Table 4 shows the mean differences for the IGDs persistence group. This group experienced an increase in motor impulsivity and agreeableness and a decrease in family partnership in T2 compared to T1.

Table 5 gives the difference of means of the different variables included in the study for the IGDs remission group. The results showed that the participants in this group had a decrease in IGDs, anxiety, hostility, and family partnership levels, displaying greater emotional stability over time.

### 3.4. Analysis of the T1 Variables That Predict IGDs in T2

A logistic regression analysis was applied to identify which variables classified adolescents with and without IGDs. Gender and type of school were controlled. The results indicated that the IGDs group were mainly male, spent more time playing video games during the week, had higher levels of depression, and were characterized by lower agreeableness and conscientiousness (Table 6). These variables explained 28% of the variance of IGDs although the percentage of correctly classified cases was only 12.5% for the IGDs group and 98.8% for the without IGDs group.

## 4. Discussion

The main objective of this study was to identify individual and interpersonal factors that could be involved in the incidence, persistence, and remission of IGDs in adolescents. The results showed that the incidence, persistence, and remission rates were 6%, 2.7%, and 4.2%, respectively. Regarding the first hypothesis, it is confirmed that there is an increase in incidence rates from 6.73% in adolescents who met the IGDs criteria in T1 to 8.73% who met the IGD criteria in T2. These results coincide with previous studies indicating that IGDs incidence rates range between 1.4% and 9.4% [63]. Longitudinal studies found that 10% of adolescents with moderate levels of IGDs increase these symptoms over time [33]. In our study, an increase in IGD symptoms was also observed in the incidence group. Online video games seem to have a high addictive potential, so vulnerable people can develop addiction because of frequent use [36]. However, remission rates were higher than persistence rates, so gaming behavior could be transitory in a specific evolutionary period and remit by itself over time, as suggested by other researchers [5]. Several factors, such as the duration of the study or age, could affect the temporal stability of the IGD [64]. In addition, there is extensive debate about gaming measurement tools due to the complex nature of the gaming experience, the heterogeneity of nomenclature and instruments, and the different methodologies used [65]. In this sense, it is important to avoid pathologizing normal gaming behaviors [66].

In line with the previous literature, significant relationships between male gender and IGDs have also been found in T1 and T2, confirming part of the second hypothesis. Men are more likely to develop addictive behaviors, such as gaming or substance abuse [33,67]. Additionally, private school students showed greater use of online video games in T1 and T2 than public school students. Perhaps this result is due to private school students’ higher socioeconomic level compared to public school students, which allows them greater access to the Internet and various technological devices. However, this result should be analyzed in greater depth since the socioeconomic level of the families was not recorded.

On the other hand, the second hypothesis about the relationship between individual, interpersonal, and contextual variables and IGDs was partially confirmed. The time spent playing video games during T1 was positively associated with IGDs in T1 and T2, as expected in the second hypothesis. Previous studies have shown that time spent on video games is associated with greater IGDs symptoms [68] and that pathological players compared to non-pathological players spend twice as much time playing, an average of six hours of online gaming per day [14,38]. Although it has also been found that the time spent gaming in itself does not affect mental health problems, vulnerable children who are poorly regulated are more likely to dedicate more time to video games [69].

In addition, IGDs were associated with emotional symptoms (anxiety and depression) and with greater hostility and impulsivity over time. This result could be related to the desire to escape from the real world to avoid negative feelings [70]. Online games could provide a virtual world in which players can escape and forget their worries in real life. In addition, previous studies suggest that adolescents with attention problems and impulsivity may have more difficulties redirecting their behavior toward other tasks and goals [71], which could expose them to the risk of developing IGDs [72]. Moreover, online games provide a world in which hostility can be expressed without restriction, allowing a space in which adolescents can manifest their aggressiveness in a way that would not be lawful in the real world [22]. It has, however, also been found that aggressive games do not in themselves seem to affect the mental health of young people [73].

The agreeableness and conscientiousness personality dimensions were negatively related to IGDs in T1 and T2, but no significant relationships were found between low extraversion and IGDs. Extraversion and agreeableness were previously found to be inversely related to IGDs [74]. People who are low in agreeableness are characterized by independence, skepticism, irritability, lack of trust in others, and the preference for individual work over cooperation. In this sense, non-cooperative online games could be preferred by people with feelings of independence [75]. In our study, emotional stability showed a significant negative association with IGDs in T1, but this association was no longer significant in T2. Perhaps adolescents gain greater emotional stability over time, and hence, this association between neuroticism and IGDs did not appear in T2. Another study found that IGDs were positively associated with neuroticism and negatively with conscientiousness [20]. Conscientiousness involves self-discipline, commitment to personal goals, and a sense of obedience and order that seems to be absent in young people who abuse the use of video games [76]. Furthermore, in our study, agreeableness, conscientiousness, and emotional stability were lower in the IGDs group than in the group without IGDs although no significant effects for the group x time interaction were found.

Several variables of family functioning were also inversely related to IGDs in T1, but these associations disappear or decrease in magnitude in T2: only negative relationships were maintained between IGDs and family adaptability and partnership. Adolescents with IGDs often live in broken family contexts [77] and could seek support and resources online. However, these resources may involve the risk of developing IGDs [78]. Family cohesion, parental supervision of video game use, and social activities with parents have been negatively associated with IGDs and could be considered protective factors for addiction [36]. A review of longitudinal studies found that a consequence of IGDs was poor parental relationships [64].

Regarding the third hypothesis, it was confirmed that the individual and interpersonal factors associated with incidence, persistence, or remission varied depending on the development pattern of the IGDs. In adolescents who developed IGDs over time (incidence group), the severity of the IGDs increased considerably. Moreover, the IGDs were found to be associated with emotional problems and low family affection. Consistent with this result, a previous study observed that youth who became pathological gamers experienced increasing levels of depression and anxiety [33,38,64]. Additionally, adolescents with depressive symptoms spent an excessive amount of time playing video games to achieve a feeling of satisfaction [79]. On the other hand, the persistence of IGDs was linked to greater time spent playing video games (two hours a day during the week and around five and a half hours on weekends), higher motor impulsivity, agreeableness, and low family partnership. In previous studies, the most impulsive players were more likely to develop IGDs [14,71,80]. This result provides some support to the theoretical proposal that suggests IGDs be considered as an impulse control disorder [81]. In addition, relationships with parents appear to play a crucial role in preventing youth from engaging in abusive video game behavior [64,82,83]. In the group of adolescents for whom the IGDs remitted (remission group), a decrease in anxiety and hostility as well as an increase in the emotional stability were observed. Players with greater emotional stability tended to perceive the real world as less threatening and stressful and were able to abandon the virtual world and online games that were presented as more controllable and safer situations [22,84]. In the same way, adolescents who were able to reduce excess video game play were found to have lower scores in anxiety and hostility [80]. This decrease in hostility could be a protective factor of IGDs. Since this finding could be associated with the genre of video games, future studies should analyze the content of video games to identify which ones would have the greatest addictive power. Higher video game addiction levels were found in children who play action, shooter, and racing games than those who did not [85].

Finally, the fourth hypothesis was partially confirmed. The only individual variables associated with greater vulnerability to experiencing IGDs over time were increased depression and decreased agreeableness and conscientiousness as well as being male and spending more time playing video games during the week. Several studies coincide in highlighting the important role of depressive symptoms in being predisposed to the development of IGDs [15,48]. Furthermore, a recent meta-analysis suggests that conscientiousness is the personality trait that most explains IGDs [23]. However, there is a debate about the role of time spent playing in the gaming disorder [86], among other reasons, because some people can play for a long time (e.g., eSports professional players) without developing IGDs. In our study, the OR values for time spent video gaming both during the week and on the weekend are very close to the threshold. This fact indicates that time spent gaming has little importance in video game addiction. This result has been shown in previous research [87]. A tentative explanation could be that the high prevalence of excessive screen time, often observed in adolescents, may result from changes in society over the last decades (major access to television, greater use of computers and the Internet) rather than addiction itself.

This study has some limitations that must be considered. First, the generalizability of the results is limited since the sample was not representative of Spanish adolescents. Furthermore, the lack of information on other sociodemographic variables, such as the socioeconomic or educational level of the families, could influence the generalization of the results. Second, the classification of the IGDs was not based on clinical interviews but rather self-report measures. Perhaps the level of evolutionary development could hamper introspection when answering the different questions, which could imply a possible bias in the data. Third, the number of young people with IGDs could be under- or overestimated due to the high dropout rate and the fact that the follow-up period was only nine months, and diagnostic criteria must be met for a continuous period of twelve months. Fourth, satisfaction with family functioning was measured by self-report. In later studies, information from third parties, such as parents and teachers, could be collected as well. In addition, analysis of family educational models and use of the Internet through observational records could be necessary. Fifth, the playing time may not be an objective variable about the extent to which gaming interferes in the daily lives of adolescents because adolescents attend school for a large part of the day, which prevents them from spending as much time gaming as they would like. A future line could assess whether all available free time is devoted to playing and whether certain affective states in specific day phases trigger the game. Finally, the data in our study were collected prior to the onset of the COVID-19 pandemic. It has been reported that the pandemic has had a continued substantial impact on psychological symptoms and disorders, addictions, and health behavior [88]. In addition, individuals have spent considerable periods of time online and have been more likely to develop a behavioral addiction [89]. During the lockdown period, 50.8% of young people reported increased gaming behavior as a coping mechanism against stress, whereas 14.6% reported a decrease [90]. However, gaming is not always problematic and can reduce loneliness, promote socialization, and reduce stress during the COVID-19 pandemic [91]. Moreover, it was also found that adolescents who presented anxiety and depressive symptoms before COVID-19 were more vulnerable to experiencing higher IGDs after COVID-19 [48]. Thus, both before and after the pandemic, it is still necessary to query the factors that increase or decrease gaming and identify which adolescents who start or persist in gaming behavior may be more vulnerable and experience adverse consequences. In addition, it is important to recognize that online gaming could have beneficial effects and to know the characteristics of adolescents for whom gaming becomes a pleasant activity limited to a specific life period and that remits over time.

Despite these limitations, this research is one of the few longitudinal studies that include individual and interpersonal variables that may influence the incidence, persistence, and remission of IGDs. The findings may be useful for the design of specific prevention strategies aimed at adolescents both at school and within the family. The improvement of emotional well-being in young people should be considered when planning prevention strategies. Moreover, family adaptation is suggested through an intervention in which parents are instructed in the proportion of emotional support for creating barriers to avoid IGDs. In addition, we propose the development of tools to promote a good use of video games that function as entertainment and for educational purposes. Previous studies have shown that if adolescents learn how to manage their free time in a balanced way and have rich and diverse extracurricular activities, the risk of excess Internet use and immersion in negative emotion would decrease [92]. Of special importance is that preventive programs be implemented at the earliest possible age to minimize the exacerbating effects on emotional symptoms as well as on the various social and family consequences discussed.

Future research in this field should address four fundamental aspects: first, to develop a greater number of longitudinal studies over a longer period to better understand the temporal dimension of IGDs; second, to identify the type of content of online games that elucidate differences in the addictive potential of specific Internet applications based on sociodemographic variables and psychosocial symptoms; third, to develop models that make it possible to establish causal relationships that clarify whether the factors analyzed are a cause or consequence of addiction; and finally, although in our study, age was not related to IGDs, it could be of interest to analyze the emotional, cognitive, and family functioning of adolescents and differences in the development of the various behavioral addictions at different stages of adolescence.

## 5. Conclusions

In sum, the results suggest that adolescents with a psychological profile characterized by emotional problems, impulsivity, and family dysfunctions are more vulnerable to developing and maintaining IGDs. Conversely, emotional stability, conscientiousness, and a good family adjustment could function as protective factors against IGDs.

## Figures and Tables

**Table 1:** Descriptive statistics and correlations for Time 1 and Time 2 (*n* = 550).

	Time 1			Time 2		
Variables	x	SD	IGDs *r*	x	SD	IGDs *r*
Gender	__	__	0.42 ***	__	__	0.44 ***
Age	13.43	1.23	−0.04	__	__	−0.07
School type	__	__	0.13 **	__	__	0.10 *
Anxiety	6.13	3.48	0.24 ***	6.39	3.82	0.11 **
Depression	3.24	2.64	0.15 ***	3.50	3.03	0.17 ***
Extraversion	9.82	2.86	0.01	9.82	2.90	−0.07
Agreeableness	10.97	2.24	−0.23 ***	10.81	2.34	−0.13 **
Conscientiousness	10.41	2.62	−0.18 ***	10.29	2.67	−0.23 ***
Emotional stability	9.67	2.73	−0.17 ***	9.43	2.86	−0.04
Openness to experience	10.90	2.51	−0.11 *	10.77	2.62	−0.07
Motor impulsivity	9.24	3.22	0.16 ***	9.78	3.53	0.20 ***
Attentional impulsivity	11.22	3.28	0.25 ***	11.29	3.21	0.21 ***
Non-planning impulsivity	11.58	3.36	0.05	11.41	3.25	0.02
Hostility	6.07	6.25	0.16 ***	6.48	7.01	0.13 **
Family adaptability	1.81	0.45	−0.19 ***	1.77	0.47	−0.09 *
Family growth	1.55	0.62	−0.11 **	1.52	0.65	−0.07
Family partnership	1.54	0.63	−0.06	1.47	0.66	−0.09 *
Family resolve	1.65	0.57	−0.19 ***	1.61	0.61	−0.04
Family affection	1.92	0.32	−0.10 *	1.90	0.36	−0.05

Note: * *p* < 0.05, ** *p* < 0.01, *** *p* < 0.001.

**Table 2:** Mean differences between the study variables between-group and within-group.

Variables	without IGDs				with IGDs				Group X Time		Time		Group	
	Time 1		Time 2		Time 1		Time 2							
	x	SD	x	SD	x	SD	x	SD	F	η^2^	F	η^2^	F	η^2^
Anxiety	6	3.43	6.23	3.81	7.56	3.75	8.01	3.62	0.35	0.001	6.12 *	0.011	16.50 ***	0.029
Depression	3.14	2.60	3.34	2.93	4.29	2.81	5.19	3.49	1.81	0.003	2.98 ^†^	0.005	19.70 ***	0.035
Extraversion	9.82	2.84	9.85	2.90	9.81	3.16	9.52	2.88	0.26	0.000	0.04	0.000	0.22	0.000
Agreeableness	11.10	2.22	10.91	2.33	9.67	2.01	9.68	2.25	0.34	0.001	0.12	0.000	19.29 ***	0.034
Conscientiousness	10.57	2.56	10.43	2.61	8.73	2.74	8.83	2.88	1.04	0.002	1.76	0.003	23.58 ***	0.041
Emotional stability	9.75	2.75	9.54	2.90	8.86	2.42	8.90	2.40	0.49	0.001	0.08	0.000	6.12 *	0.011
Openness to experience	10.92	2.48	10.75	2.63	10.72	2.84	10.90	2.51	1.32	0.002	1.74	0.003	0.06	0.000
Motor impulsivity	9.11	3.16	9.60	3.48	10.54	3.51	11.73	3.47	2.12	0.004	3.38 ^†^	0.006	15.90 ***	0.028
Attentional impulsivity	11.06	3.21	11.13	3.18	12.93	3.55	12.96	3.15	0.002	0.000	0.17	0.000	17.71 ***	0.031
Non-planning impulsivity	11.56	3.37	11.42	3.26	11.76	3.32	11.34	3.17	0.246	0.000	0.32	0.001	0.12	0.000
Hostility	5.84	6.24	6.16	6.93	8.46	5.86	9.77	7.01	1.15	0.002	2.14	0.004	14.77 ***	0.026
Family adaptability	1.82	0.44	1.78	0.46	1.69	0.51	1.67	0.52	0.24	0.000	0.14	0.000	4.66 *	0.008
Family growth	1.56	0.62	1.53	0.65	1.44	0.68	1.44	0.65	0.09	0.000	0.00	0.000	1.98 **	0.004
Family partnership	1.55	0.64	1.48	0.65	1.48	0.58	1.40	0.64	0.00	0.000	0.10	0.000	0.96	0.002
Family resolve	1.66	0.55	1.62	0.60	1.48	0.71	1.50	0.68	0.20	0.000	0.15	0.000	4.82 *	0.009
Family affection	1.92	0.31	1.90	0.34	1.88	0.39	1.79	0.50	1.56	0.003	0.80	0.001	3.86 *	0.007

Note: ^†^ *p* < 0.10, * *p* < 0.05, ** *p* < 0.01, *** *p* < 0.001.

**Table 3:** Difference of means in the variables evaluated between T1 and T2 for the incidence group.

Variables	Time 1		Time 2			
	x	SD	x	SD	t	d
IGDs	2.59	1.36	5.55	0.67	−11.54 ***	−2.76
Anxiety	6.91	3.55	8.08	3.71	−1.77 ^†^	−0.32
Depression	4.00	2.35	5.36	3.60	−2.24 *	−0.45
Extraversion	9.70	2.98	9.45	3.01	0.51	0.08
Agreeableness	9.97	201	9.39	2.35	1.63	0.27
Conscientiousness	9.03	2.77	8.81	2.72	0.39	0.08
Emotional stability	8.88	2.17	8.56	2.08	0.76	0.15
Openness to experience	10.95	2.73	10.72	2.76	0.38	0.08
Motor impulsivity	10.46	3.55	11.48	3.37	−1.49	−0.29
Attentional impulsivity	12.50	3.59	12.85	3.29	−0.56	−0.10
Non-planning impulsivity	11.47	3.15	11.31	3.25	0.24	0.05
Hostility	8.00	5.55	9.76	7.29	−1.54	−0.27
Family adaptability	1.79	0.42	1.70	0.53	1.00	0.19
Family growth	1.45	0.67	1.42	0.66	0.20	0.05
Family partnership	1.45	0.62	1.45	0.67	0.00	0.00
Family resolve	1.61	0.66	1.52	0.67	0.77	0.14
Family affection	1.91	0.29	1.76	0.50	1.72 ^†^	0.37

Note: ^†^ *p* < 0.10, * *p* < 0.05, *** *p* < 0.001.

**Table 4:** Difference of means in the variables evaluated between T1 and T2 for the persistence group.

Variables	Time 1		Time 2			
	x	SD	x	SD	t	d
IGDs	6.22	0.88	5.80	1.15	1.00	0.41
Anxiety	9.00	3.91	7.87	3.54	1.20	0.30
Depression	4.95	3.68	4.80	3.32	0.21	0.04
Extraversion	10.07	3.61	9.67	2.66	0.41	0.13
Agreeableness	9.00	1.93	10.33	1.91	−1.98 ^†^	−0.69
Conscientiousness	8.07	2.66	8.87	3.29	−0.97	−0.27
Emotional stability	8.80	2.98	9.93	2.84	−1.17	−0.39
Openness to experience	10.20	3.10	11.29	1.86	−1.42	−0.43
Motor impulsivity	10.73	3.54	12.27	3.73	−2.27 *	−0.42
Attentional impulsivity	13.87	3.38	13.20	2.93	0.89	0.21
Non-planning impulsivity	12.41	3.71	11.41	3.10	0.90	0.29
Hostility	9.47	6.56	9.80	6.60	−0.20	−0.05
Family adaptability	1.47	0.64	1.60	0.51	−0.56	−0.22
Family growth	1.40	0.74	1.47	0.64	−0.44	−0.10
Family partnership	1.53	0.52	1.27	0.59	2.26 **	0.47
Family resolve	1.20	0.77	1.47	0.74	−1.29	−0.36
Family affection	1.80	0.56	1.87	0.52	−1.00	−0.13

Note: ^†^ *p* < 0.10, * *p* < 0.05, ** *p* < 0.01.

**Table 5:** Difference of means in the variables evaluated between T1 and T2 for the remission group.

	Time 1		Time 2			
Variables	x	SD	x	SD	t	d
IGDs	5.67	1.09	2.48	1.46	8.92 ***	2.47
Anxiety	7.13	3.45	5.70	3.98	2.24 *	0.38
Depression	4.91	3.03	5.04	3.84	−0.14	−0.04
Extraversion	9.00	3.03	9.04	3.32	−0.08	−0.01
Agreeableness	9.23	2.87	10.09	2.59	−1.33	−0.31
Conscientiousness	9.70	2.29	9.15	2.47	0.94	0.23
Emotional stability	7.43	2.79	8.55	2.79	−1.83 ^†^	−0.40
Openness to experience	9.91	2.66	9.48	2.50	1.22	0.17
Motor impulsivity	10.61	3.84	10.90	3.41	−0.54	−0.08
Attentional impulsivity	12.82	2.84	12.23	2.50	0.81	0.22
Non-planning impulsivity	12.19	3.43	12.08	3.11	0.14	0.03
Hostility	9.84	7.97	7.39	6.83	2.24 *	0.33
Family adaptability	1.64	0.64	1.70	0.56	−0.32	−0.10
Family growth	1.46	0.78	1.26	0.75	1.03	0.26
Family partnership	1.55	0.66	1.22	0.74	1.51	0.47
Family resolve	1.46	0.66	1.43	0.66	0.16	0.05
Family affection	1.86	0.46	1.83	0.49	0.30	0.06

Note: ^†^ *p* < 0.10, * *p* < 0.05, *** *p* < 0.001.

**Table 6:** Logistic regression analysis of the predictive variables of the T1 on IGDs in T2.

Variables	β	Wald X^2^	OR	IC 95% (Low, High)
Time spent playing video games during the week	0.005	6.56 **	1.01	(1.00, 1.01)
Time spent playing video games on the weekend	0.002	1.78	1.00	(0.99, 1.00)
Gender	0.849	3.47 ^†^	0.43	(0.18, 1.05)
School type	0.506	1.77	0.60	(0.29, 1.27)
Anxiety	0.060	1.09	1.06	(0.95, 1.20)
Depression	0.118	2.96 ^†^	1.13	(0.98, 1.29)
Extraversion	0.002	0.001	1.04	(0.90, 1.20)
Agreeableness	−0.196	5.19 *	0.82	(0.69, 0.97)
Conscientiousness	−0.180	6.55 **	0.84	(0.73, 0.96)
Emotional stability	0.042	0.28	1.04	(0.89, 1.22)
Openness to experience	0.119	2.29	1.13	(0.97, 1.31)
Motor impulsivity	0.002	0.001	1.00	(0.88, 1.14)
Attentional impulsivity	0.092	1.95	1.10	(0.96, 1.25)
Non-planning impulsivity	0.092	2.58	0.91	(0.82, 1.02)
Hostility	0.015	0.17	0.99	(0.92, 1.06)
Family adaptability	0.081	0.04	0.92	(0.41, 2.07)
Family growth	0.142	0.21	0.87	(0.47, 1.59)
Family partnership	0.126.	0.18	1.14	(0.63, 2.03)
Family resolve	0.305	1.11	0.74	(0.42, 1.30)
Family affection	0.130	0.06	1.14	(0.40, 3.26)

Note: ^†^ *p* < 0.10, * *p* < 0.05, ** *p* < 0.01.

## Data Availability

The data presented in this study are available on request from the corresponding author. The data are not publicly available due to the participants were minors.
